# Facial and Vocal Expressions During Clinical Interviews Suggest an Emotional Modulation Paradox in Borderline Personality Disorder: An Explorative Study

**DOI:** 10.3389/fpsyt.2021.628397

**Published:** 2021-03-24

**Authors:** Javier Villanueva-Valle, José-Luis Díaz, Said Jiménez, Andrés Rodríguez-Delgado, Iván Arango de Montis, Areli León-Bernal, Edgar Miranda-Terres, Jairo Muñoz-Delgado

**Affiliations:** ^1^Facultad de Psicología, Universidad Nacional Autónoma de México (UNAM), Mexico City, Mexico; ^2^Dirección de Investigaciones en Neurociencias, Instituto Nacional de Psiquiatría Ramón de la Fuente Muñiz (INPRFM), Mexico City, Mexico; ^3^Facultad de Medicina Universidad Nacional Autónoma de México (UNAM), Mexico City, Mexico; ^4^Clínica de Trastornos de Personalidad, Instituto Nacional de Psiquiatría Ramón de la Fuente Muñiz (INPRFM), Mexico City, Mexico

**Keywords:** prosody, emotional conflict, speech characteristics, FaceReader, PRAAT, social engagement system, exploratory network analysis, multilevel models

## Abstract

Videotape recordings obtained during an initial and conventional psychiatric interview were used to assess possible emotional differences in facial expressions and acoustic parameters of the voice between Borderline Personality Disorder (BPD) female patients and matched controls. The incidence of seven basic emotion expressions, emotional valence, heart rate, and vocal frequency (*f0*), and intensity (dB) of the discourse adjectives and interjections were determined through the application of computational software to the visual (FaceReader) and sound (PRAAT) tracks of the videotape recordings. The extensive data obtained were analyzed by three statistical strategies: linear multilevel modeling, correlation matrices, and exploratory network analysis. In comparison with healthy controls, BPD patients express a third less *sadness* and show a higher number of positive correlations (14 vs. 8) and a cluster of related nodes among the prosodic parameters and the facial expressions of *anger, disgust*, and *contempt*. In contrast, control subjects showed negative or null correlations between such facial expressions and prosodic parameters. It seems feasible that BPD patients restrain the facial expression of specific emotions in an attempt to achieve social acceptance. Moreover, the confluence of prosodic and facial expressions of negative emotions reflects a sympathetic activation which is opposed to the social engagement system. Such BPD imbalance reflects an emotional alteration and a dysfunctional behavioral strategy that may constitute a useful biobehavioral indicator of the severity and clinical course of the disorder. This face/voice/heart rate emotional expression assessment (EMEX) may be used in the search for reliable biobehavioral correlates of other psychopathological conditions.

## Introduction

Expressing, detecting, and evaluating emotions are crucial social and cognitive skills for behavioral adaptation. Human emotions are mainly expressed in facial, postural, verbal, or vocal behaviors and usually involve physiological correlates, such as heart rate. These expressions manifest a variety of subjective states and constitute objective behaviors that can be recorded and closely scrutinized. There is extensive evidence of the salient role that facial expressions, verbal and vocal parameters play in the communication of basic emotions among human beings (1). In face-to-face human encounters, the simultaneous emission of facial and vocal expressions expands the information that results in the recognition and attribution of emotional states that played an important role in human evolution.

Dysfunctions in the recognition of facial expressions have been observed in several major psychiatric disorders but there is scarce information concerning the emission of emotional expressions. It is feasible that specific deviations of facial and vocal expressions of emotion may be of diagnostic value in psychiatry. The combined face/voice communication channel constitutes a parameter with reliable psychometric properties allowing for the discrimination among depressive disorders (2), and atypical emotional expressions have been reported in schizophrenia, depression, and autism spectrum disorders (3). In the same review, it was stressed that most of the currently available methods to assess emotional expression have not been validated in clinical settings, a desirable condition to ascertain their diagnostic value.

Customary office settings facilitate the recording of facial and vocal behaviors that allow for multiple and punctual analyses of emotional reactions. The availability of computerized discrimination and quantification systems of facial and vocal expressions of emotion (4) constitutes an opportunity to assess such expressions in psychiatric patients during clinical interviews. In eating disorders and schizophrenia, a software called multiple-fusion-layer based ensemble classifier of stacked autoencoder (MESAE) that analyzes the range of emotional expression according to facial arousal has been applied (5). The facial and vocal expression of Borderline Personality Disorder (BPD) patients during a psychiatric clinical interview has not been studied and is likely to provide valuable diagnostic information.

BPD diagnosis requires the presence of five of the following criteria: (1) Fear of abandonment, (2) Unstable and intensive relationships with rapid changes between idealization and derogation, (3) Identity disturbances, (4) Impulsivity and risk-taking behaviors, (5) Recurrent suicidal behavior, (6) Threat of committing suicide and self-injurious behaviors, (7) Emotional instability, (8) Feelings of emptiness, (9) Inappropriate anger, (10) Uncontrolled aggression, (11) Stress-dependent paranoid ideation or dissociative symptoms (6).

From an evolutionary and behavioral ecology perspective, BPD can be understood as a condition that emerges from the interaction of genetic vulnerabilities with experiences of early emotional adversity, unresponsiveness of attachment figures, trauma, and abuse. This confluence might hinder the person's expectation regarding future resource availability in terms of social interactions, such that individuals would tend to maximize short-term benefits from interpersonal relationships and undertake a “fast” Life History Strategy (LHS). Fast LHS has been characterized by heightened threat sensitivity, low tolerance of frustration, poor executive control, novelty-seeking, impulsivity, risk proneness, low parenting effort, unstable intimate relationships, high cortisol levels, reduced heart rate variability, and early sexual maturation (7, 8). An inadequate assessment and interpretation of perceived other's emotions may distort behavioral and vocal expressions and foster a deterioration of interpersonal relationships (9).

Emotional vocalizations are adaptive for social species; in humans, speech development provides multiple prosodic means to express emotions and convey affective connotations during face-to-face human communication (10). Adjectives and interjections constitute emotionally loaded vocal expressions (11), where adjectives provide effective verbal evaluations and interjections constitute brief and intense vocal expressions as it occurs in other animal vocal emissions.

The autonomic nervous system (ANS) regulates the emotional interpretation of environmental and internal stimuli, particularly those evoking fear and anger (12). ANS not only contributes to the hedonic valence of sensations and percepts, but the organization of the emotional expression involving physiological, motor, and social interactions, such as fight or flight response (sympathetic), fainting, “playing dead” (dorsal vagal), or affiliative behaviors (ventral vagal) (13).

Considering the reported emotional dysregulation in BPD patients (14), it seems justified to analyze possible differences in the facial expressions of the basic emotions, the emission and prosodic properties of adjectives and interjections, as well as autonomic correlates. Therefore, the present study aimed to compare the incidence of the seven basic emotion expressions, the emotional valence, arousal, heart rate, and the incidence and vocal parameters of adjectives and interjections between five BPD patients and matched controls. We hypothesized that BPD patients exert a subliminal control of their facial and vocal emotional expression where autonomic modulation plays an inferable role. The relatively small patient sample is justified in terms of the rigorous diagnostic and matched control criteria employed and the extensive and meticulous analyses of the behavioral and autonomic variables recorded by precise computational means.

## Materials and Methods

The present study was conducted in two stages. First, an audiovisual recording was obtained during an initial clinical interview of five BPD female patients and five female controls matched by age and educational level [age: t_(8)_ = 0.3, *p* = 0.77; education: X^2^ = 2.58, *p* = 0.98]. Afterward, by the use of the FaceReader and PRAAT programs applied to the video and audio recordings, the incidence of the seven basic emotion expressions, the emotional valence, arousal, heart rate, and the incidence and vocal parameters of adjectives and interjections were analyzed.

### Participants

#### Patients

Five female patients diagnosed with BPD by DSM-IV criteria were selected after their first admission to the Personality Disorder Clinic of the *INPRFM* in Mexico City. BPD was diagnosed by two board-certified psychiatrists when patients met DSM-IV criteria (15). Subjects were excluded if they presented a comorbid diagnosis of antisocial personality disorder, acute psychotic episode, acute manic episode, active eating disorder, substance abuse disorder (except for nicotine), neurodevelopmental disorder, and a depressive episode of moderate or high severity {>17} in accord with the Hamilton Rating Scale for Depression (16). Hamilton scores were: 9, 1, 1, 4, 4 and corresponding pharmacological treatments were as follows: Fluoxetine 60mg/day + Quetiapine 100mg/day, none, Escitalopram 30mg/day, Fluoxetine 20mg/day, Fluoxetine 20mg/day + Topiramate 50mg/day.

All of the patients have a history of traumatic experiences, three of sexual abuse childhood, one has a comorbidity of Post Traumatic Stress Disorder (PTSD), another of complex trauma and physical violence.

All patients completed the Clinical Interview for DSM-IV Axis II Personality Disorders Self-Report Screening Questionnaire (SCID-II/PQ) to explore all personality disorders, including BPD (17). As recommended, we used a cutoff point of >5 to establish the BPD diagnosis. Two subjects met 15 criteria, another two 11 criteria, and one, 13. The overall personality disorder diagnosis agreement reported with the use of the SCID-II/PQ vs. the SCID II interview (K = 0.75) is adequate (18). The average age of participants was 28.8 years (S.D. = 6.4, range 22–39). The patients had High School (4 subjects) or Bachelor's degrees (1 subject). The scoring on BEST measures was the following: 33, 46, 55, 53, and 42. According to the number of BPD criteria met in the SCID-II/PQ and the scores in the BEST questionnaire it can be said that the group of patients was relatively homogeneous. The interviews lasted 30 min, but only an average of 11.42 min was recorded for analyses. Neither the greetings nor the farewells were recorded for both groups.

#### Controls

Five women with an average age of 29.6 years (S.D. = 5.18, range 24–38) were selected to participate in the study after an invitation on the Internet was issued and answered. The control group had completed a Bachelor's to a Master's degree. The clinical instrument SCID-II/PQ was applied following a two-tiered procedure. First, the respondents completed the digitized questionnaire; the five women in the group who did not disclose any disorder on Axis II were subjected to an individual face-to-face interview similar to the therapeutic intervention received by the patients. The average length of the interview for the control group was 17.00 min.

#### Ethics

The study was conducted following the general principles stipulated in the Declaration of Helsinki. Even though it is usually difficult to obtain reliable recordings of clinical interviews because of confidentiality constraints, in this case, once the objectives and procedures of the investigation were explained, it was possible to achieve careful informed consent from legally proficient patients and controls concerning their anonymity and the value of the clinical research. The project was approved by the Ethics and Research Committees of *INPRFM*.

#### Procedure

The interviews were held in a 4 × 2 meter, well-lit, and quiet consulting room of the *INPRFM* Personality Disorders Clinic. Both patient and control subjects had a session conducted by a single male clinician (EMT) certified in the Acceptance and Commitment Therapy (ACT) technique of Hayes et al. (19). The psychological intervention was designed to meet the requirements of the ACT to provide a homogeneous and comfortable environment in which the interview and recordings took place. At the beginning of each session, participants were guided in a 5-min mindfulness exercise, and subsequently, the interview was directed to detect recent and present cognitive distortions, unpleasant emotions, and/or recent problematic behaviors. Once a salient problem was detected, the interview was directed to explore the symptoms and difficulties in-depth. The subject's facial and vocal expressions were recorded with a Canon XA10-HD camera placed 1.5 meters in front of each patient focusing on the face.

#### Facial Expression of Emotion

The FaceReader 7 software was used to obtain and quantify facial expressions of emotion every 0.04 hundredths of a second (time-lapse frame) of the recording video. This software detects and classifies seven facial expressions of the basic emotions: happiness, sadness, anger, surprise, fear, disgust, contempt, plus a neutral expression (20). The software establishes the emotional valence as an index obtained by calculating the intensity of positive emotion (happiness or surprise) minus the negative emotion with the highest intensity (21). Arousal of 20 Action Units of the Facial Action Coding System (FACS) indicates whether the patient is active {+1} or not active {0}. Heart rate was acquired by photoplethysmography (face luminance depending on vasodilatation).

#### Audio

The audio signal was extracted from the video recordings and converted to WAV format (Waveform Audio File) with a sample rate of 44,000 Hz, 8-bit resolution, and monophonic signal. The acoustic parameters of the voice were analyzed from these audio recordings with the PRAAT computer program (22) as the fundamental frequency (*f0*) expressed in Hz, and loudness in decibels (dB). Sampling time was set at 0.01 hundredths of a second. The adjectives and interjections within the verbal discourse were defined according to the *Diccionario de la Real Academia Española*, and then searched and selected with the PRAAT program. The audio is played by the program, and when an adjective or an interjection was recognized and selected by one of the authors, the program delivers the *f0* and the dB values. Lastly, the duration of the interjections was synchronized with the FaceReader 7 timeline to obtain the emotional valence, arousal, and heart rate together with the timeline data of the fundamental frequency and the decibels of the voice.

#### Statistical Analysis

Since the FaceReader software records a frame every 0.04 hundredth of a second, each subject has about ±17,000 of repeated measurements of facial expression. To evaluate the differences in emotional variables between BPD and control samples, linear multilevel models (or linear mixed-effects models) were performed to consider the correlated structure of the observations. Data is said to be correlated because repeated measurements of the same participant are expected to be more similar to each other than measurements among participants (23). Multilevel models were fitted for each emotion to estimate the differences between groups using what is called *fixed effects*, and at the same time, to estimate the individual variability and the correlated structure of the data we use random intercepts by subject (24). It is essential to mention that performing a two-sample *t*-test would lead to biased estimates of the differences between the groups so that only multilevel models could estimate the parameters in an unbiased way with acceptable rates of type I and type II errors (24, 25).

As an exploratory analysis, the correlation between emotional facial expressions and vocal indicators was calculated for each group using the Pearson method considering a statistical significance at the 0.05 level, and to visualize the associations between the variables in each group, with the correlations which absolute values were set above the 0.4 level, two exploratory networks were built using the force-directed layout algorithm implemented in the “ggraph” package of R (26).

The data were preprocessed and extracted using the Python language for scientific computing (27). Then, all models were programmed in R using the lme4 and lmerTest packages to test the hypotheses of whether the differences between the samples were different from zero (28). Finally, all *p*-values were adjusted using the False Discovery Rate (FDR) to reduce type I errors due to multiple dependent comparisons (29).

## Results

### Facial Expression of Emotion

Table 1 shows the results of the FaceReader analysis of the 7 basic emotions in the BPD and the control groups. Even though there were differences between the two groups in surprise and disgust, the only significant disparity was found in the facial feature of sadness, where the patients expressed less than half the amount of this emotion compared to the control group (0.30 vs. 0.051, *p* < 0.02). The emotional valence showed a significant difference between the groups, tending to zero in the patients (−0.114 vs. −0.002 in a −1 to +1 range; Table 1). To justify this preliminary result, a power analysis using 1000 Monte Carlo simulations in the case of the emotion of sadness between the groups was made with our sample of 10 subjects. A power of 95.8% (IC 95% = 94.36, 96.96) was obtained which is well above the recommended 80% as acceptable. According to the power curve analysis (Supplementary Material), a seven subject sample would be enough to reach such a level in the design of further studies using the same or very similar temporal conditions.

**Table 1:** Facial expression of emotion frequency.

	**Groups**				**95% CI**			
**Variable**	**Controls**		**BPD**		**Lower**	**Upper**	**Statistic**	***P*-adjust**
	**Mean**	**SD**	**Mean**	**SD**				
Neutral	0.586	0.197	0.652	0.203	−0.073	0.151	0.67	0.68
Happy	0.053	0.129	0.075	0.168	−0.004	0.075	1.72	0.34
Sad	0.13	0.228	0.051	0.103	−0.119	−0.04	−3.92	0.02^*^
Angry	0.019	0.04	0.011	0.024	−0.016	0	−1.83	0.34
Surprised	0.106	0.18	0.067	0.136	−0.089	0.021	−1.19	0.49
Scared	0.034	0.062	0.026	0.055	−0.019	0.002	−1.64	0.34
Disgusted	0.012	0.028	0.006	0.015	−0.014	0.002	−1.39	0.49
Contempt	0.012	0.035	0.015	0.04	−0.006	0.009	0.44	0.78
Valence	−0.114	0.262	−0.002	0.214	0.066	0.187	4.05	0.02^*^
Arousal	0.354	0.184	0.348	0.163	−0.06	0.068	0.12	0.91
Heart rate	68	8.759	70	9.332	−3.12	9.92	1.01	0.53

* *p < 0.05*.

### Prosody

Concerning the expression of adjectives and interjections, a total of 177 adjectives were obtained from both groups, 108 (61%) uttered by the BPD group, and 69 (39%) by the controls, while the proportion of interjections was higher in the control group 126 (60%) than in patients 83 (40%). The proportion tests were highly significant for both adjectives (X^2^ = 16.32, *p* < 0.001) and interjections (X^2^ = 12.88, *p* < 0.001). No significant differences were found in the fundamental frequencies and decibels of adjectives and interjections of both groups (Table 2).

**Table 2:** Acoustic parameters of voice frequency.

	**Groups**				**95% CI**			
**Variable**	**Controls**		**BPD**		**Lower**	**Upper**	**Statistic**	***P*-adjust**
	**Mean**	**SD**	**Mean**	**SD**				
Adjectives (*f0*)	177	67.3	197	73.6	−26.34	51.99	0.72	0.68
Interjections (*f0*)	172	66.5	177	90	−17.17	20.8	0.19	0.9
Adjectives (dB)	61.5	5.91	63.6	7.39	−2.75	8.66	1.12	0.49
Interjections (dB)	61.8	5.93	61.4	7.4	−4.71	2.68	−0.6	0.68

*f0 = Fundamental frequency in Hertz*.

*dB = Intensity in decibels*.

### Correlation Matrix

**Figure 1:**
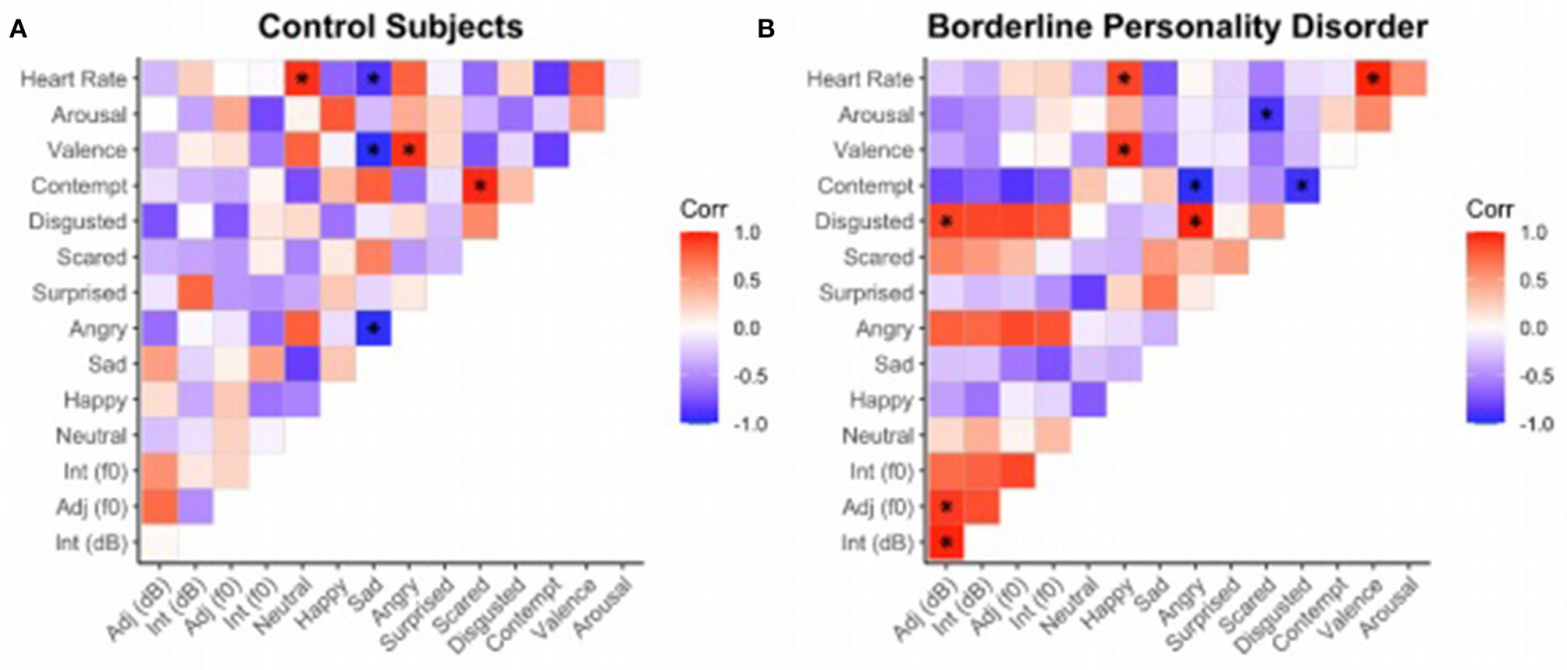
Correlation matrices established among emotional variables (Neutral, Happy, Sad, Angry, Surprised, Disgusted, Valence, Arousal) and acoustic parameters (*f0, dB*) of controls **(A)** and patients **(B)**. The correlation index on each cell is displayed in a gradient of colors where red represents a positive correlation (0 > r > 1); blue, a negative one (−1 < r <0); and white the absence of revelent correlation.

Figure 1 shows the correlation matrices among the heart rate, arousal, valence, basic emotions, and acoustic parameters in controls (left) and BPD patients (right). Compared to the control group, patients show a higher number of correlations, especially positive ones (8 vs. 14), among the elements of prosody and facial expression of some negative emotions. Specifically, a positive correlation was observed in patients between facial expressions of disgust and anger, and the acoustical parameters of adjectives and interjections, both in decibels and in fundamental frequency. This correlation was not present in the control group. Additionally, some correlations are opposite in control's and patient's groups; particularly, the following pairs are negative in controls and positive in patients: heart rate and happiness, disgust and adjectives (*f0-*dB), anger and adjectives (dB), and anger and interjections (*f0*).

### Network Results

**Figure 2:**
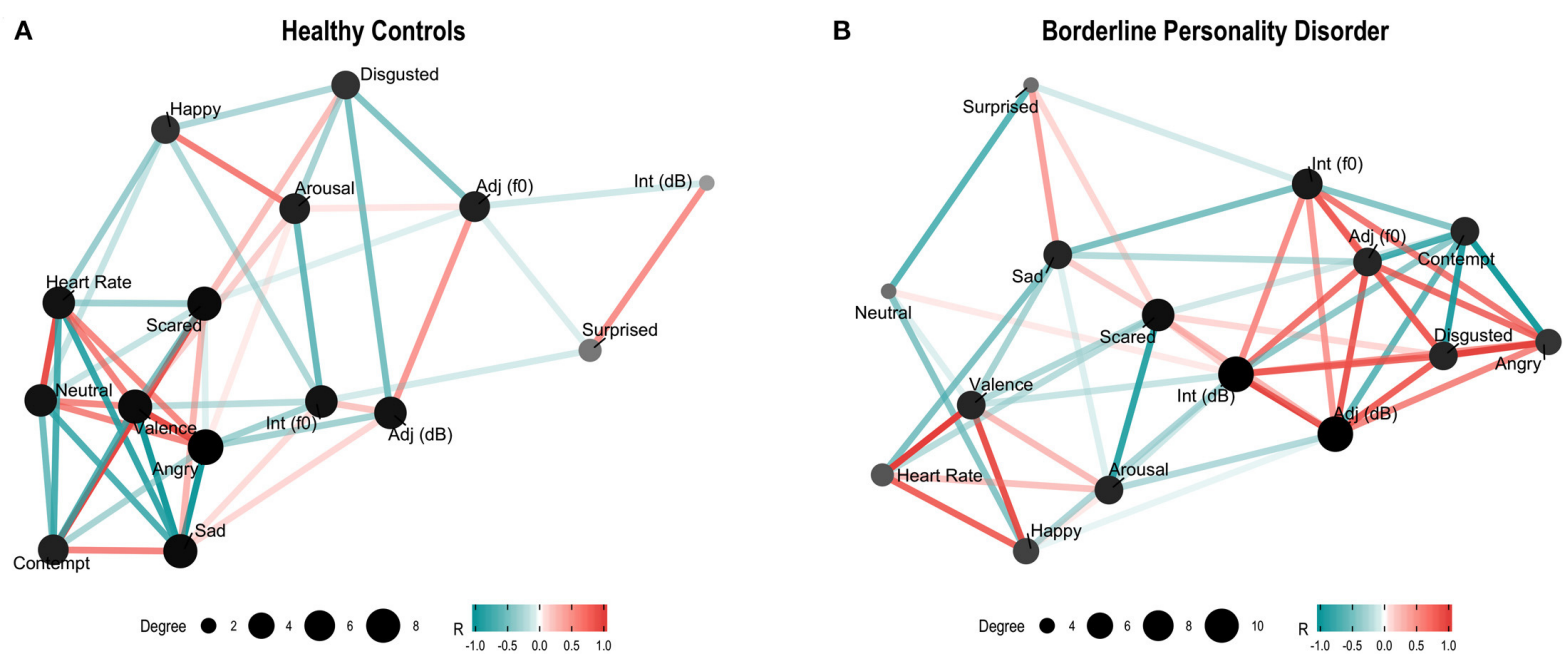
Controls **(A)** and patients **(B)** *force-directed layout* networks among facial expressions, acoustic parameters (*f0–dB*), valence, heart rate, and arousal. The nodes represent recorded variables; their size and color intensity reflect the number of correlations: bigger and darker nodes represent a greater number of relations. Edges connecting pairs of nodes reflect the strength of their correlation. Color intensity (red for positive and blue for negative) reflects the correlation strength.

The two exploratory networks built from the force-directed layout algorithm and implemented in the “ggraph” package of R are shown in Figure 2. Panel A on the left side depicts the network obtained for the control group. The group of nodes dominating the net in terms of the number of connections for each variable (dot size) and intensity of correlations among the variables (positive correlations in red and negative correlations in blue) includes the negative emotions of Anger, Sadness, Contempt, and Fear. Panel B on the right shows the network corresponding to the BPD patients. The set of nodes dominating the network in terms of the intense interconnections includes the vocal variables of intensity (dB) and frequency (*f0*) of interjections and adjectives together with facial expressions of the negative emotions of Anger, Disgust, and Contempt. The second set of nodes appears in the lower left of this network characterized by positive relations among Happy, Valence, and Heart Rate. The main difference between the two networks consists in the set of interconnections among acoustic and facial parameters of repulse emotions depicted in the patient's group which is absent in the controls, where the relation of acoustic and facial expressions is weak and does not include Contempt.

## Discussion

Even though there were only 10 subjects for the between-groups comparison it is possible to justify these preliminary results in terms of the extensive and meticulous recording and statistical procedures. During the interview, each subject was recorded by the FaceReader software every 0.04 s for an average period of 11.4 min resulting in ±17,000 data points. The advantage of such longitudinal measurements by subject is that it diminishes the variance produced by individual differences, resulting in more sensitive and uniform data, greater confidence of between-subject comparisons, and the detection of real effects with fewer and extensively analyzed subjects.

The emotionality of psychiatric patients is usually evaluated during clinical interviews based on the sensibility and expertise of a trained psychiatrist, sometimes aided by self-reports and other indirect measures of emotion. Quantitative indicators of emotion are of particular interest because of the relevance that affective alterations play in mental disease. The present study constitutes an attempt to detect objective and quantitative indicators of mental disorders that may constitute biomarkers of clinically characterized psychopathological conditions. This is the first time that facial and vocal expressions are measured and compared between BPD patients and matched controls using a computational software of audiovisual recordings obtained during a clinical psychiatric interview. It is also the first attempt to synchronize facial and vocal recordings obtained from the same source allowing the analysis of congruence between both expressions of emotion. Specifically, we attempted to find differences in the facial expressions of the basic emotions and the prosodic voice parameters of adjectives and interjections recorded in videotapes.

The main significant result of the facial behavior showed that patients express more than a third less sadness compared to controls. We consider two possible explanations for this preliminary result: patients may feel or express less sadness. It has been reported that patients with mood disorders attempt to muffle their emotions (30) and suppress their expression (31). In states of emotional dysregulation, which can be observed in BPD patients, they undertake “functional strategies” to suppress or avoid their emotions, usually resulting in greater anxiety and dysfunction (32). In turn, the prolonged use of emotional regulation strategies in search of social acceptance leads to maladaptive behaviors (31) and altered interpretations of the perceived behaviors of others (33). Reduced emotion was reported in patients with anorexia nervosa (34); therefore, the reduced expression of sadness observed in our results favors this suppression hypothesis. On the other hand, our result of a neutral emotional valence in patients, compared to the control group which showed a tendency toward a negative valence, favors the possibility of an emotional disconnection among emotions, emotional labels, and expressions. This possibility is strengthened by previous results that report that BPD patients may exhibit diverse degrees of alexithymia (35, 36). These BPD patients seem to fail in interpreting their own and other's emotions while being highly responsive to other's emotional expressions (14, 37). Moreover, it is likely that concealing sadness, displaying anger, disgust, and contempt may be the consequence of feeling threatened by the male interviewer, especially since all of the patients have a history of trauma and abuse. Inactivating the social engagement system in favor of a fight or flight mode seems congruent with some elements of the Fast Life History Strategy suggested for these patients (7, 8).

Even though the acoustic parameters of tone and volume of adjectives and interjections were similar between the two groups, the emission frequency was different, the number of adjectives was higher in patients while interjections were more common in the control subjects. Prior studies reported that subjects with BPD vocalize more adjectives with positive valence with the supposed purpose of favoring the acceptance and empathy of others (38–40).

If BPD patients constrain the exclamations manifesting direct emotions, then the lower expression of interjections could be explained in terms of the suppression hypothesis. It is also possible that in the context of an initial interview, the control volunteers express more interjections in response to the therapist's interventions (7, 41).

The positive correlation among the expressions of anger and disgust with the *f0* of adjectives and interjections, and between the expression of happiness and heart rate, may be related to the emotional hyperreactivity and ANS instability, particularly a high sympathetic tone, reported in BPD (42, 43). From an evolutionary perspective, an attachment over-reactivity correlated with an increased sympathetic tone has been suggested in BPD patients; such hyperactivation constitutes an adaptive intent that leads to the establishment of fast and intense social interactions, frequently burdened with negative emotions (7).

Thus, the intensification of discomfort-suggesting signals, such as facial expressions of anger and disgust, underlined with adjectives and interjections, function as poorly adaptive strategies to signal a need for help. Such an interactive scheme may come especially into play during an initial clinical interview where patients accentuate the solicitation of assistance. The negative correlation between the expression of disgust and prosodic variables may reflect the attempt to reduce the emphasis of this emotion during the clinical interaction, and thereby increase the possibilities of acceptance and support.

Since one of the main clinical traits in BPD patients is negative affectivity (44), we suggest that this feature may manifest in the positive interconnections observed between the prosodic voice features and negative facial gestures. Even though such facial/vocal outflow of affective information during a face-to-face interaction facilitates the recognition of the emotional state by the receptor or decoder (45), this multichannel emission of negative and rejecting emotions in BPD patients communicates an internal activation of the fight/flight response and an interpersonal distancing endeavor. Moreover, these signals reflect an activation of the sympathetic nervous system associated with increased arousal and motility which is opposite to the social engagement system involving the ventral parasympathetic branch (42).

Our exploratory results suggest that even though in the context of a clinical interview BPD patients seek empathy, rapport, and assistance, their diminished expression of sadness, their confluence of prosodic and facial expressions of negative emotions, and their sympathetic signals communicate a very different or even opposite motivational state. Such paradox places the patient in an interactional situation that may contribute to interpersonal conflict, which is another prominent BPD feature. As it has been suggested in studies of synchrony in BPD patients, “their alertness in social situations may hinder them to fully engage non-verbally” (46). Our initial results may add to the understanding of alterations in the quality of relationships in this patients. These findings can shed light on a possible non-verbal, emotional, and behavioral maladaptive strategy to be noticed by the clinician and monitored not only as a marker of the personality disorder but also if modified in the direction of the activation of the social engagement system, as a clinical indicator of treatment outcome.

Even though the present study has a limited sample size due to the strict enrollment criteria employed, the precise and laborious measurements of facial and vocal signals make these methods and findings potentially relevant to the understanding, detection, and evaluation of BPD and other mental disorders. This face/voice/heart rate emotional expression assessment (EMEX) may be used in the search for reliable biobehavioral correlates of other psychopathological conditions. Future studies should be undertaken to improve the confidence level and reduce the variability of the results. Similar studies in larger and culturally varied populations will be necessary to confirm if the present data constitute objective and reliable BPD indicators. Considering the controversy about the universality of facial and prosodic expression of emotion it would be important to conduct further research on this topic in other social cultural and linguistic scenarios.

## Data Availability Statement

The raw data supporting the conclusions of this article will be made available by the authors, without undue reservation.

## Ethics Statement

The project was approved by the Ethics and Research Committee of Instituto Nacional de Psiquiatría Ramón de la Fuente Muñiz. The patients/participants provided their written informed consent to participate in this study.

## Author Contributions

JV-V and JM-D: concept, design. EM-T: clinician interview. EM-T, JV-V, and SJ: data acquisition. JV-V and SJ: statistical analysis. JV-V, J-LD, SJ, IA, AR-D, AL-B, and JM-D: figures. JV-V, J-LD, SJ, IA, AR-D, AL-B, and JM-D: manuscript writing. All authors contributed to the article and approved the submitted version.

## Conflict of Interest

The authors declare that the research was conducted in the absence of any commercial or financial relationships that could be construed as a potential conflict of interest.
