# Supplementary figures and images for: Facial and Vocal Expressions During Clinical Interviews Suggest an Emotional Modulation Paradox in Borderline Personality Disorder: An Explorative Study
Source: Front Psychiatry. 2021 Mar 24;12:628397. doi: 10.3389/fpsyt.2021.628397 (PMC8024539; doi:10.3389/fpsyt.2021.628397)

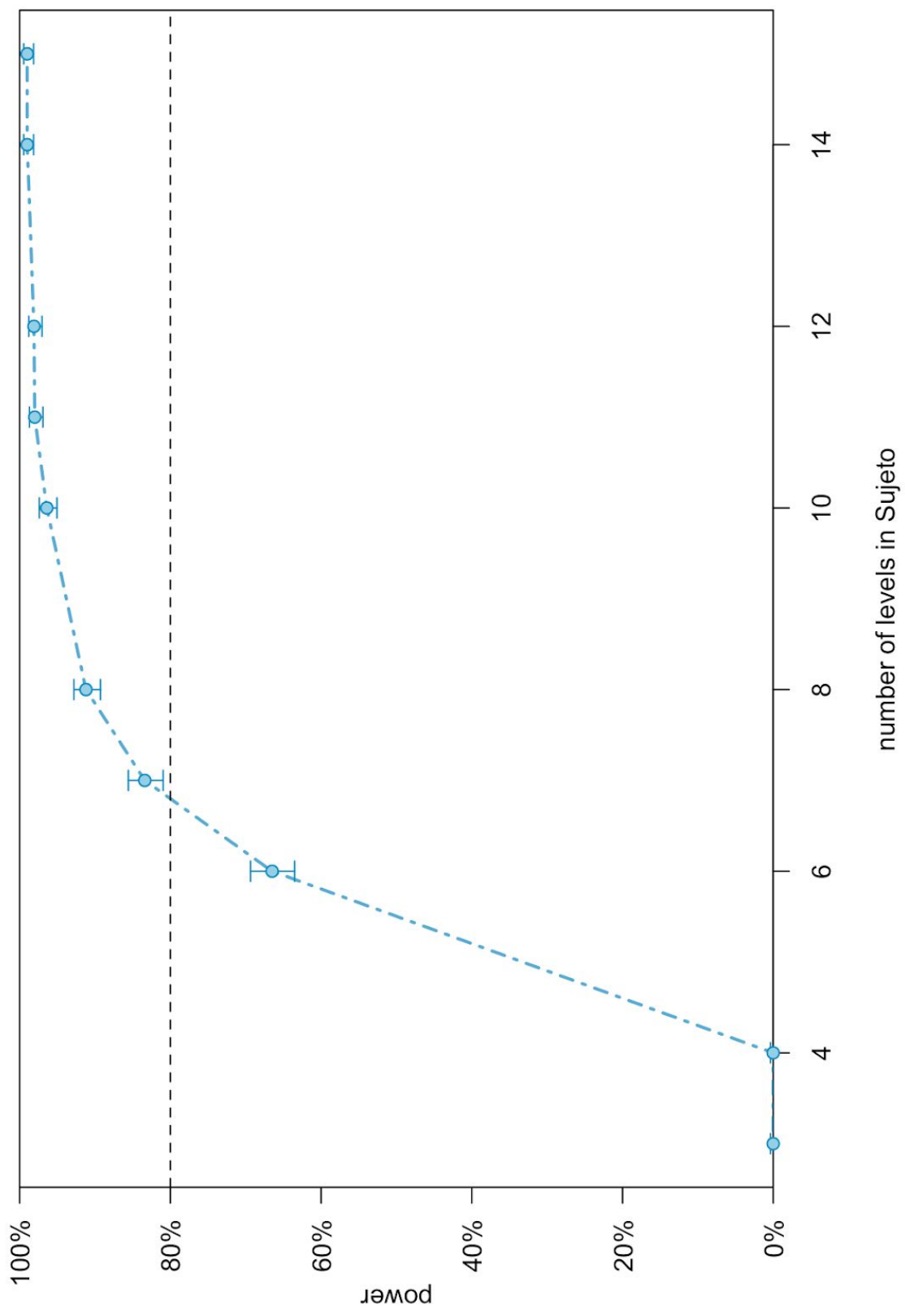

Supplement: Supplementary file 1 [file Data_Sheet_1.PDF]
